# "Nurses Are Shamefully Underpaid": A Step on the Way

**Published:** 1918-10-05

**Authors:** 


### "NURSES ARE SHAMEFULLY UNDERPAID."

### A Step on the Way.

The correspondence, official and unofficial, which has followed Major Chapple's letter to The Hospital is distinctly satisfactory, but whether from his point of view or not I do not know. I have no desire to trespass the borders of this controversy nor to confuse the issue, as I am interested generally rather than in a particular institution. As a matter of fact I do not regard the London Hospital as a greater sinner than a few others, although I was the first to protest against its policy of "farming" its nurses and absorbing in its revenues the profits made through their instrumentality. Whether other hospitals find it more difficult to obtain the necessary funds, or whether they are less efficiently organised, is not in evidence. The fact remains that those which refrain from hiring out their staffs have not claimed to be more liberal than the "London." Some who do, however, are known to be so. I had a very specific object in view when I called attention to the "farming" system, and this was to demonstrate beyond dispute that nurses are underpaid, and that their earnings, whether hired out or not, are spent without their consent on public philanthropy. It has never been put before a nurse probationer that she is expected to contribute from her earnings to the cost of nursing the nation's sick poor.

First Fruits of Pioneering Work.

During the months which have elapsed since I first ventilated this matter a tremendous advance has been made. Readers will recollect that Lord Knutsford's argument was "my bond." The nurse knew beforehand what her conditions of employment were to be, and, being a free agent, she was under no obligation to accept them. In other words, every agreement must necessarily be fair simply because it is an agreement. This attitude is now officially abandoned, if the House Governor of the "London" speaks with authority. "I am in entire agreement," he says, "that nurses are shamefully underpaid." So far as I am aware this is the first occasion on record that such an announcement has emanated from a British hospital board, and it is all to the credit of the "London" that it should be first in the field with such a confession. Such I regard as the first-fruits of the pioneering work of this Journal in the field of economics, and I desire, therefore, again to accentuate the importance of nurses realising the value of the press as an instrument of reform. Union, articulation, and better conditions of employment comprise the gospel preached to nurses by The Hospital as opposed to the eternal question of legislation, which can achieve little more than the compiling of a list of names.

A Tangible Goal to Reach.

Let it not be supposed that I am counting these steps in the way of progress by way of boasting. My object is to cause every nurse in the country to realise that there is a tangible goal to reach, and that it can be reached only by united movement. I want them to understand that in their own efforts, and not in the efforts of six or seven hundred members of Parliament, is their hope of salvation. For thirty barren years, by devious paths, they have been led in pursuit of a will-o'-the-wisp. What they really want is a charter of employment. For this The Hospital has been labouring, and I think we have got very well on the way when a leading institution in the Metropolis is obliged to admit that the whole profession is "shamefully underpaid."

Mr. Morris's Illuminating Letter.

Mr. Morris's letter to the Editor is otherwise illuminating. "Why should I blush," he inquires, "when I admit that the profit on nursing the sick rich is spent on nursing the sick poor?" May I congratulate the writer on his frankness, and may I at the same time adopt the Hibernian method of making reply by asking another question? **Do the British public expect their sick poor to be nursed at the expense of the women who regard nursing as their vocation? Mark you, it is not "the sick rich" who are footing the bill. A sick rich person pays the market price for the services of a trained nurse, and she "delivers the goods." This is an honourable commercial or professional transaction, and neither party is under any** *complement* **to the other. It is the nurse who pays the forfeit, and I venture to ask Mr. Morris whether he thinks it is in accord with a high standard of ethics to call on hard-working women, earning their living, to discharge what is in reality a national obligation?** I do not know how far Mr. Morris has travelled on this road, nor to what extent Lord Knutsford's chairman words express his personal convictions. It is, however, very gratifying to learn from Mr. Morris that Lord Knutsford himself, and other members of the London Hospital board, are opposed to profiteering.

Why the British Public will stop the Sweating of Nurses.

I do not believe that the true facts have ever yet been put before the British public. I am not out to condemn British hospitals or the members of British hospital boards. Their achievements are above criticism. Their time, their labour, and their purses have been placed at the disposal of the nation without stint and without display. All that I charge them with is that they have forgotten the workers. With the highest intentions they have "sweated" and "exploited" the nurses. Of this there is no room for doubt, and I use these words without reserve. The British public is rich and does not call for such sacrifice. **If the case is properly presented there is not the smallest doubt that the necessary funds will be forthcoming.**

I Know my Nurse well. She has not Taken a Vow of Poverty.

The concluding sentence of Mr. Morris's letter sheds a world of light on the attitude of hospital boards. Nurses themselves, he says, regard their work as a mission, and not simply as a trade. Herein lies the secret of the fallacy. I venture to say that I know my nurse as well as Mr. Morris. She is not a woman of means, nor yet a woman who has taken a vow of poverty for the salvation of her soul. She loves her work, but she is as much a wage-earner as the physician and surgeon. She is out for a career, and if any member of the London Hospital board harbours a doubt as to whether this is true or not he has abundant means at his disposal of ascertaining the facts. The editor of the *Times* may regard his work as his "mission," but it is likewise his "trade," and nobody expects him to spend his time, his talent, and his income on the cause of the sick poor. Why should the nurse do so?

Sound the Trumpet.
Win Justice and Missionise too.

I regret that I cannot acquit nurses themselves of blame that it should be possible for the House Governor of a large hospital calmly to suggest that British nurses

**"Ierne" On Nurses Shamefully Underpaid**—(*cont.*) are blind to their personal interests, and that such vital questions as remuneration and reasonable hours of toil do not count in their scheme of life. This is the covert innuendo which he seeks to convey when he states that they regard their work as a mission and not as a trade, and it would be impossible for Mr. Morris even to hint such an absurdity but for the nurses' constant and continual silence. It is hoped that this misrepresentation will be a lesson to the whole nursing profession. *If they cannot or dare not sound the trumpet note of truth in the Board Room there is no reason why they cannot do so in the press.* In the sanctum of the Editor she can speak without fear, and with no uncertain sound.

Make clear Nurses are not without Ambition.

Every member of the profession from matron down is directly and profoundly interested in making quite clear, even if she loves her work, that she is not without ambition and the desire to partake of the emoluments of faithful service. Until the nurse emerges from her mysterious silence and makes her voice resound above the tumult she will be the victim of misrepresentation and of public philanthropy, falsely so called. IERNE.

